# Effect of Oxidant Concentration on the Oxide Layer Thickness of 304 Stainless Steel

**DOI:** 10.3390/ma17122816

**Published:** 2024-06-10

**Authors:** Kerong Wang, Haixu Liu, Ning Liu, Xiaoming Chen, Jiapeng Chen

**Affiliations:** 1 Jiangsu Key Laboratory of Precision and Micro-Manufacturing Technology, College of Mechanical and Electrical Engineering, Nanjing University of Aeronautics and Astronautics, Nanjing 210016, China; jhcwkr@163.com (K.W.); 20060847@jmi.edu.cn (X.C.); 2 Mechanical and Electrical Engineering College, Jinhua Polytechnic, Jinhua 321000, China; 3 Research Center for Advanced Micro-/Nano-Fabrication Materials, School of Chemistry and Chemical Engineering, Shanghai University of Engineering Science, Shanghai 201620, China; 4 State Key Laboratory for High Performance Tools, Zhengzhou Abrasive Grinding Research Institute Co., Ltd., Zhengzhou 450001, China; 5 State Key Laboratory of Silicon and Advanced Semiconductor Materials, Zhejiang University, Hangzhou 310027, China

**Keywords:** 304 stainless steel, nano-scratch, oxide layer, electrochemical corrosion

## Abstract

Ultra-thin 304 stainless steel can be used to flexibly display substrates after they have been subjected to chemical mechanical polishing (CMP). The thickness of the chemical oxide layer directly affects the polishing efficiency and surface quality of 304 stainless steel. In the study presented in the following paper, the thickness variation of the chemical oxide layer of 304 stainless steel was analyzed following electrochemical corrosion under different oxidant concentration conditions. Furthermore, the impact of the oxidant concentration on the grooves, chips, and scratch depth–displacement–load curves was investigated during a nano-scratching experiment. Through this process, we were able to reveal the chemical reaction mechanism between 304 stainless steel materials and oxidizers. The corrosion rate was found to be faster at 8% oxidant content. The maximum values of the scratch depth and elastic–plastic critical load were determined to be 2153 nm and 58.47 mN, respectively.

## 1. Introduction

304 stainless steel is an important material for national industry and defense development; it acts as the “blood vessels” of industry. This particular form of stainless steel is widely used in metallurgical machinery, civil and military navigation, and other industries owing to its excellent toughness, plasticity, weldability, and corrosion resistance at standard and low temperatures [1,2,3,4]. In the majority of cases, LED substrates are used as semiconductor materials. However, it is expected that ultra-thin stainless steel will replace semiconductors as the mainstream material used for LED substrates due to its high degree of perdurability. The use of stainless steel and semiconductors as LED substrates results in very high requirements for its substrate surface quality and performance, such as fewer surface and internal defects, light weight and high strength, etc. [5]. Numerous scholars have carried out in-depth research into the polishing tools, process, and mechanism of stainless steel surfaces. There is consensus among scholars that chemical mechanical polishing (CMP) is one of the most important techniques in realizing the ultra-smooth and damage-free processing of 304 stainless steel surfaces. However, CMP is a form of three-body wear; the structural and CMP properties of the stainless steel itself have a crucial impact on the yield, service performance, and service life of flexible substrates [6]. Chemical reactions between the polishing solution and workpiece during CMP generate a new oxide layer on the surface of the stainless steel and change its structure. The thickness of the oxide layer produced using different concentrations of polishing solution directly affects material removal form, removal efficiency, and surface quality.

Wang et al. [7] used the CMP method to smooth the surface of bearing steel and found that a change mass fraction of H_2_O_2_ in the polishing solution affected the performance of the reaction layer and reduced surface roughness (4 nm). Zhang et al. [8] analyzed the multiple influencing factors responsible for the CMP effects of mold steel. The mold steel (main components: Fe and FeCr) was corroded rapidly in strongly acidic polishing solutions when the pH value was altered. Finally, the process parameters were altered to achieve a super smooth surface (3.11 nm). Weng et al. [9] found that the use of H_2_O_2_ increased the hydrophilicity of the stainless steel surface, and a Fenton-type Haber–Weiss reaction occurred during the non-destructive processing of the stainless steel surface when using the CMP method. This process facilitated the removal of surface oxides. Furthermore, the use of this method reduced the stability of the oxide layer and improved the redox reaction induced by H_2_O_2_. Wu et al. [10] used the NH_2_ functional group as a complex in the CMP process of GCr15 bearing steel; the surface film of bearing steel in alkaline (pH = 10) environments demonstrates better corrosion and wear resistance than in acidic (pH = 4) environments. In addition, roughness average (Ra) gradually decreased with increasing pH value. The results of the aforementioned studies indicate that the oxide layer on the surface of the workpiece directly affects the surface quality and performance of the polished workpiece during the CMP process. However, these studies lack sufficient data on the thickness of the oxide layer; thus, analysis of the evolution and quantification of oxide thickness during the CMP process is an indispensable step in ultra-precision machining.

At present, authors of studies on the thickness of the oxide layer focus on qualitative analyses [11,12,13,14]; thus far, researchers have been unable to visually indicate whether the thickness of the oxide layer is completely destroyed following the CMP process. Therefore, establishing an effective method for determining the thickness of the oxide layer will have a crucial impact on the CMP effect and material removal method used. In comparison, nano-scratching is a commonly used method for the detection of the continuous process of change in mechanical properties at the microscopic scale. Material properties, such as the elastic–plastic deformation domain, brittle fracture, and surface scratch resistance, can be tested using the nano-scratching method. Wang et al. [15] proposed a new method of nanoscale deformation to fabricate nanostructures; in their study, the phase transition and defect formation mechanism of the Si sheet during the scribing process were analyzed, and the width and depth of the scratches were determined to be 456 nm and 33 nm, respectively. Zhang et al. [16] investigated the effect of scribing speed on material structure by using a new method of high-speed scribing with a nanoscale depth of cut and determined the final width and depth of residual scribing at the onset of chip formation to be 288 to 316 nm and 49 to 62 nm, respectively. Cheng et al. [17] analyzed the effect of Ra and scratch depth on the corrosion behavior of stainless steel using the droplet method and showed that the occurrence of pitting corrosion was related to the destruction of an important component in the passivation film through the use of surface analysis techniques. Kuromoto et al. [18] found nucleated cracks on the surface of austenitic stainless steel during cathodic charging at room temperature and used the nano-scratch method to verify whether the hydrogenation of stainless steel alters the plastic and elastic behavior. The results of their study show that the scratch morphology depends strongly on the crack distribution and grain orientation, with the results helping to enhance our understanding of the hydrogen embrittlement problem in austenitic stainless steel. Although the aforementioned researchers conducted nano-scratch studies, the removal behavior and structural transformation/damage of the materials were mainly investigated through the use of the nano-scratch method; very few studies have been conducted to determine the thickness of the oxide layer by studying the way in which the material is removed via nano-scratching.

Therefore, to address this problem, in the present study, the authors first mechanically polished 304 stainless steel to reach the same initial state, then electrochemically etched the surface of the steel using different concentrations of oxidizing agents, and finally carried out the variable load scratching experiment. The elastic–plastic transformation critical load and critical depth value (aberration point) of 304 stainless steel were analyzed using the elastic mechanics theory to investigate the stainless steel etching mechanism. The effect of change in oxidizer concentration on the thickness of the oxide layer on the surface of 304 stainless steel was investigated based on the depth of the aberration point.

## 2. Materials and Methods

**Figure 1:**
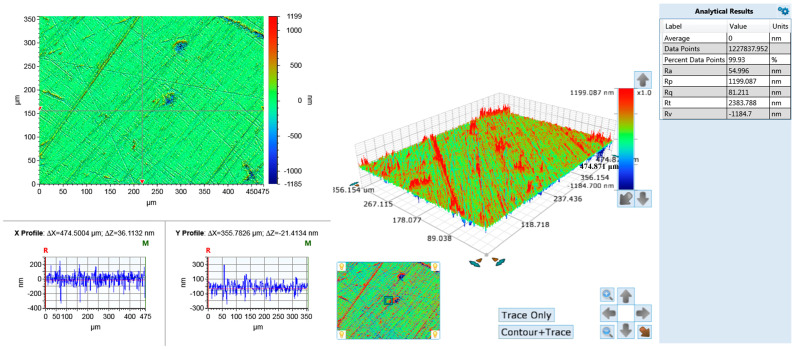
The initial surface morphology of 304 stainless steel.

The test samples comprised 304 stainless steel sheets (several samples) with a radius of 25.2 mm and a thickness of 2 mm, acquired from Shenzhen Ruikai Mould Co. (Shenzhen, China). The main components of stainless steel detected via energy dispersive spectroscopy mainly contain Cr, Mn, Fe, and Ni. Detailed composition data are shown in Table 1. The stainless steel sheets were ground prior to the experiment; the abrasive material used was Al_2_O_3_ particles, and it was dispersed with glycerol. The specific grinding process parameters are shown in Table 2. The surface morphology of the stainless steel following grinding is shown in Figure 1. The surface roughness (Ra) values of the samples were all in the range of 53–55 nm.

Four corrosion solutions with different oxidant (FeCl_3_) concentrations were prepared using the controlled variable method, wherein the oxidant content was 2%, 4%, 6%, and 8%. The stainless steel sheet was placed in the corrosion solution, and the voltage during the electrochemical corrosion experiment was maintained between 0 and 10 V, the operating current was stabilized at 145 mA, the temperature of the corrosive solution was 60°, and the solution stirring speed was 25 r/min. The pH value of the corrosion solution was controlled using oxalic acid (pH = 2), and the duration of the experiment was 20 min.

**Figure 2:**
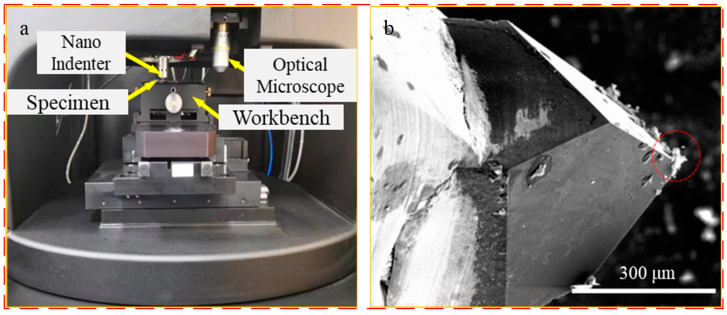
Nano-scratch test system: (**a**) nano-scratch equipment and (**b**) nano-indenter.

**Figure 3:**
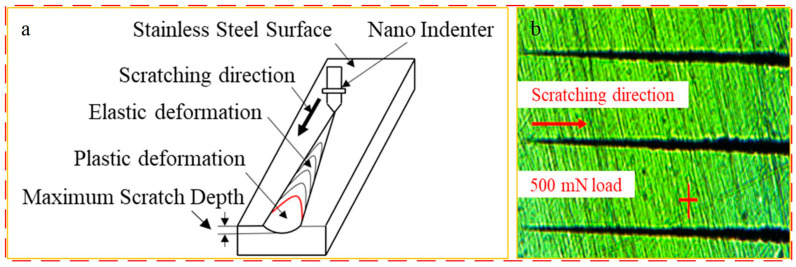
Nano-scratching process: (**a**) schematic diagram of the scratching process and (**b**) scratching surface morphology.

The nano-scratch tests were all performed using the nanoindentation meter G200MTS system manufactured by KLA, Silicon Valley, CA, USA, and the system is shown in Figure 2. Each set of scratch tests was performed three times to ensure the accuracy of the test results, and the scratching process and results are shown in Figure 3. The 3D morphology of the stainless steel surface was obtained using a Bruker 3D surface profiler (Contour GT-K with a vertical resolution of 0.1 nm) manufactured by Bruker, Billerica, MA, USA. A scanning electron microscope (SEM) (S-3400N, manufactured by Hitachi, Tokyo, Japan) was used to observe the surface morphology before and after scratching, and an energy dispersive spectroscopy (EDS) system (Oxford Inca 250, manufactured by Oxford Instruments, Oxford, UK) was used to determine the surface element content. In this experiment, EDS was performed with a working distance of 9.6 mm, a loading voltage of 20 kV, a spot diameter of 4 mm, and a scanning time of 10 s. The surface layer composition of the stainless steel was analyzed before and after electrochemical corrosion using an XRD system (Bruker D8 DISCOVER, Billerica, MA, USA). The scratch test was carried out with a constant load, applying normal load values of 0–500 mN, approaching a surface speed of 20 nm/s, a scratch speed of 30 μm/s, a scratch width of 0–50 μm, a scratch length of 500 μm, and a distance of 70 μm between two neighboring scratches. The surface scratch morphology is shown in Figure 3b.

## 3. Results and Discussion

### 3.1. Scratching Characteristics

**Figure 4:**
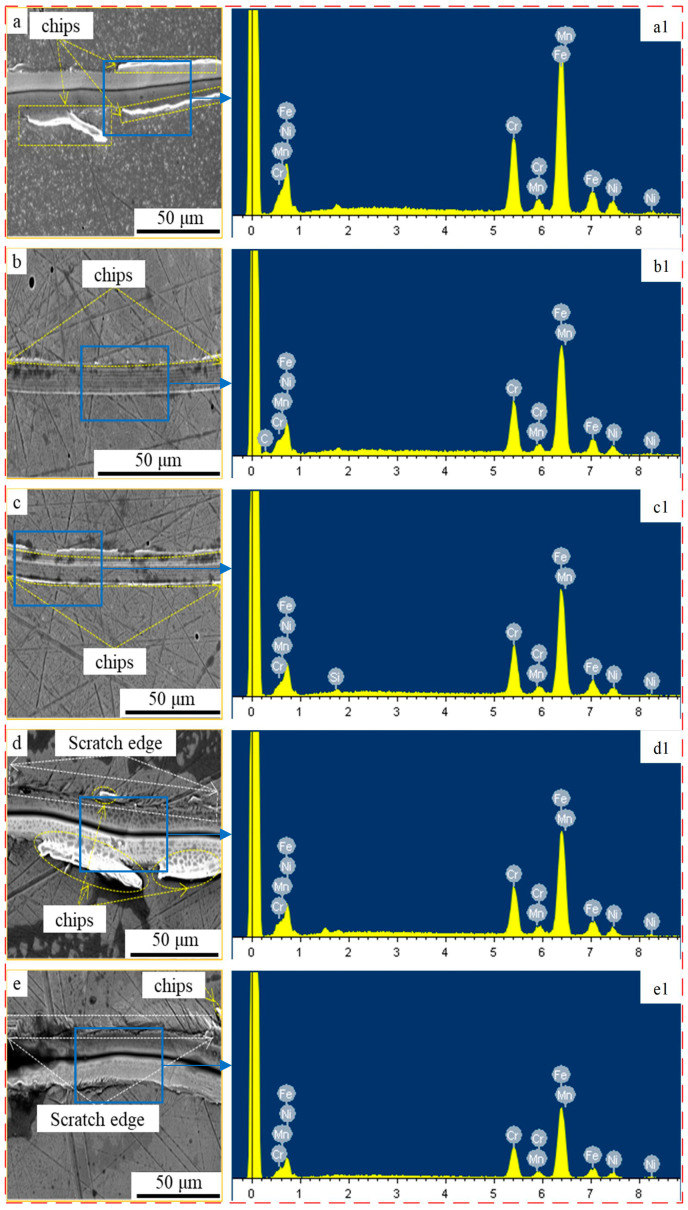
Scratching morphology and energy dispersion spectrum under the effect of electrochemical corrosion: (**a**,**a1**): oxidizer concentration 0%, (**b**,**b1**): oxidizer concentration 2%, (**c**,**c1**): oxidizer concentration 4%, (**d**,**d1**): oxidizer concentration 6%, and (**e**,**e1**): oxidizer concentration 8%.

Figure 4 displays the nano-scratch morphology of the stainless steel following electrochemical corrosion under varying oxidant concentrations. Figure 4a shows the SEM morphology of the initial stainless steel surface after scribing. It can be seen that there are band chips and cracks at the edge of the scratch. This morphology is inextricably linked to the inherent properties of stainless steel. Moreover, this result suggests that the shear force exerted on the stainless steel surface during the scribing process did not reach the critical value of its fracture strength. Figure 4b–e depicts the SEM images of nano-scratched surfaces subjected to electrochemical corrosion with varying concentrations of oxidants, revealing the disappearance of scribed band chips and the emergence of plowed pear-like features on the surface. As the concentration of corrosive solution increased (Figure 4d), wide and thick chips formed once again. The chips shown in Figure 4e eventually disappeared. This was the result of the scratch experiment being conducted in the plastic domain [19,20]. As a result, the stainless steel scratch experiments did not show obvious cracking as the corrosion concentration increased. Conversely, obvious porosity can be observed on the electrochemically corroded surface due to the extremely aggressive nature of chloride ions, which can effectively promote the localized destruction of the stainless steel surface layer and produce a pitting effect [21,22]. Our results also demonstrate that FeCl_3_ as an oxidizing agent is effective at oxidizing stainless steel. Furthermore, the SEM images presented in Figure 4a–e indicate that electrochemical corrosion has a significant magnifying effect on the surface scratches of stainless steel following mechanical polishing. This finding can aid in the further examination of the quality of stainless steel surface layers.

**Figure 5:**
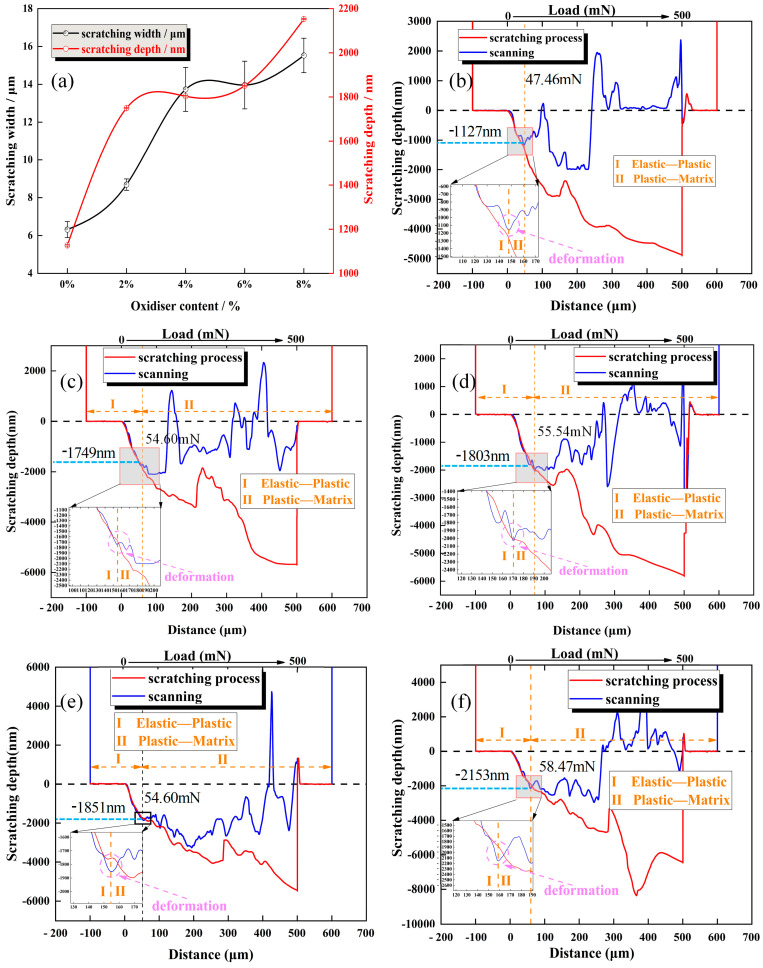
Scratching dimensions and nano-scratch depth–displacement–load curve on the 304 stainless steel surface following electrochemical corrosion with varying oxidant content: (**a**): effect of oxidizer content on scratching width and depth, (**b**): oxidizer concentration 0%, (**c**): oxidizer concentration 2%, (**d**): oxidizer concentration 4%, (**e**): oxidizer concentration 6%, and (**f**): oxidizer concentration 8%.

After an electrochemical corrosion experiment, the surface was tested using an energy-dispersive spectrometer, as presented in Figure 4a1–e1. The results indicate that as the oxidizer concentration increased, the elemental content exhibited irregular variations. Notably, as the oxidizer concentration increased from 4% to 6%, the scribed surface showed significant differences, transitioning from scratches solely on the oxide layer to scratches on both the oxide and substrate layers (Figure 4c,d). This indicates that at an oxidizer concentration of 6%, the electrochemical corrosion is intensified, leading to erosion of the stainless steel surface. In this case, the oxide layer was penetrated, scratches appeared on the surface of the stainless steel substrate, and a similar phenomenon is also observed in Figure 4e. The scratching results in Figure 5 indicate that the scribing depth is 1803 nm at an oxidizer concentration of 4%. As the oxidizer concentration increases, the scribing depth continues to increase (1851 nm and 2153 nm), and there was no significant turning point occurred in the scribing depth at an oxidizer concentration of 4%. Therefore, it can be proven that at an oxidizer concentration of 6–8%, significant erosion of the stainless steel occurs. This explains the phenomenon observed in Figure 4a1–e1, where changes in elemental content are present but not pronounced.

### 3.2. Elastic–Plastic Evolution—Softening Layer Thickness

The depth and width of scratches after electrochemical etching are shown in Figure 5a. It can be observed that the scratch dimensions increase as the concentration of oxidizer increases. This is the same result found in the above analysis of electrochemical corrosion behavior.

Depth–displacement–load curves of nano-scratches at 0%, 2%, 4%, 6%, and 8% oxidant contents are shown in Figure 5. During the scratch process, the method of material removal and the corrosion rate of oxidants on the surface of the stainless steel change. Critical depth and load during the elastic–plastic transformation of stainless steel materials were determined to explain the effect of oxidant content on the thickness of the stainless steel oxide layer. As shown in Figure 5b–f, the elastoplastic transition depths and critical loads are 1127 nm, 1749 nm, 1803 nm, 1851 nm, and 2153 nm and 47.46 mN, 54.54 mN, 55.54 mN 54.60 mN, and 58.47 mN, respectively. Upon further analysis, we found that the elastic–plastic transition value of the stainless steel was the point at which the scanning curve first distorted during the scanning process. As the oxidizer content increased, the depth of the indenter pressing vertically into the material surface also increased. The highest depth was observed at 2153 nm when the oxidizer content was 8%, as depicted in Figure 5f, and the slope of the depth–displacement–load curves increased with the increase in oxidant content, indicating a corresponding increase in the electrochemical reaction rate. Variations in the critical load significantly affect the scribing test process involved in stainless steel removal. During the scratch experiment, the initial stainless steel surface obtained a critical load value of 47.46 mN (Figure 5b). The critical load values on the erosive stainless steel surface increase slightly, however, the scratching depth values raise obviously, therefore, the softening layer formed by the oxidizer is existed. 

### 3.3. Mechanisms of Electrochemical Corrosion

**Figure 6:**
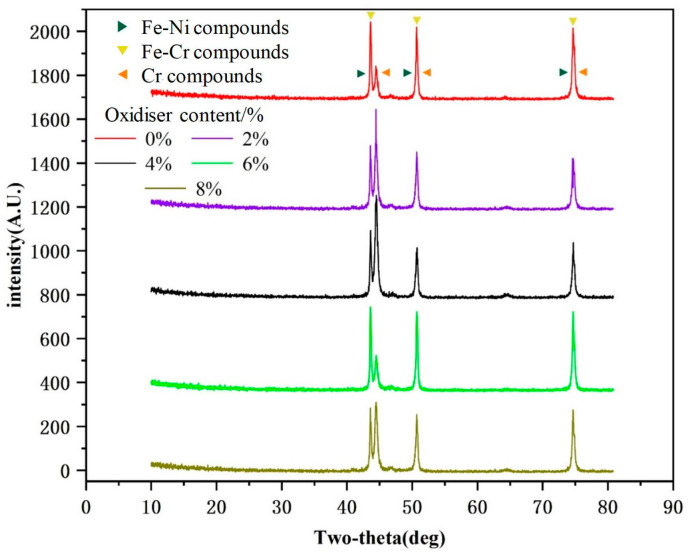
Electrochemical corrosion XRD spectra.

It is widely known that the use of stainless steel is indispensable in flexible substrates due to its excellent toughness. It would be more beneficial for those involved in the development of industrial applications to understand the material transformation mechanism that occurs during stainless steel processing [23,24]. Therefore, in the present study, the stainless steel was subjected to XRD to allow us to obtain information about the changes in the elemental content of the surface following galvanic corrosion, and the results are shown in Figure 6.

Based on the XRD pattern analysis results, it appeared that the main compounds found in the products were Fe, Cr, and Ni. This composition suggested that the basic physical structure of the stainless steel sheet remained unchanged despite changes in its other components. The results also indicated that electrochemical corrosion had not affected the stainless steel’s fundamental physical phase. The results displayed in Figure 6 show that different oxidant contents had varying degrees of influence on the XRD curve; the main reason for this observation was that different amounts of oxidants had different oxidation rates during chemical corrosion [25]. The experiment resulted in low levels of FeCl_3_ due to the low concentration of oxidant. FeCl_3_ was able to dissolve to produce H^+^ in an alkaline environment. As a result, the subsequent redox reaction had a low potential initially. However, the chemical reaction weakens during the later stage due to the high number of precipitates produced. This in turn reduced the reaction rate on the surface of the stainless steel, as shown in Equation (1) [26]. During the experiment, the pH value of the FeCl_3_ solution was in an acidic environment (pH = 2–3). Therefore, the H+ concentration in the FeCl_3_ solution increased due to the low pH value, which changed the hydrolysis reaction to an alkaline environment [27,28]. With the increase in H^+^ concentration, the decrease in Fe^3+^ concentration was inhibited, causing the reaction shown in Equation (1) to proceed to the left. This resulted in an increase in Fe^3+^ concentration and enhanced the reaction process shown in Equation (2). Simultaneously, the redox reaction of Equations (3)–(5) continued. This was the main aspect responsible for the elemental content of stainless steel not changing significantly after EDS testing was performed.

(1) $${\mathrm{Fe}}^{3+}+3H_{2}O↔Fe{\left(OH\right)}_{3}↓+\;3H^{+}$$

(2) $${2\mathrm{Fe}}^{3+}+Fe\to 3Fe^{2+}$$

(3) $$\mathrm{Fe}+2FeCl_{3}\to 3FeCl_{2}$$

(4) $$\mathrm{Cr}+{3\mathrm{FeCl}}_{3}\to {\mathrm{CrCl}}_{3}+{3\mathrm{FeCl}}_{2}$$

(5) $$\mathrm{Ni}+2FeCl_{3}\to NiCl_{2}+2FeCl_{2}$$

In an acidic environment created by FeCl_3_ as an oxidizing agent, the stainless steel would have a breakdown potential (E_b_). A transient current broke through the passivation film of the stainless steel, causing pitting corrosion. When the potential exceeded the breakdown potential (Eb), this led to an increase in the number of pitting holes on the surface of the stainless steel and the generation of new ones. This result was the same as the pitting effect that occurs on the surface after galvanic corrosion, as discussed in Section 3.1. As the content of oxidants increased, the solubility of oxidants on the surface alloy of the stainless steel decreased during the electrochemical corrosion reaction process. However, acidic environments could maximize the effect of oxidants. Due to the higher concentration of Fe^3+^, the increase in the solution reaction time and the number of complexes in the stainless steel resulted in a decrease in Fe^3+^ content in the solution. The continuous exposure of Fe element in the stainless steel resulted in an increase in Fe^2+^ ions, which elevated the Ni^2+^ and Cr^2+^ content, thus leading to the decreased corrosion resistance of the solution. The kinetics of the chemical reaction were analyzed, and it was established that the concentration of the reactants decreased during the reaction; in contrast, the concentration of the products increased and reached a maximum at a specific solution concentration. This aspect was responsible for the product’s peak value, shown in Figure 6, increasing continuously with the increase in oxidant content. The continual decrease in the concentration of the solution will cause the etching rate to decrease and ultimately stabilize at a specific value; this phenomenon demonstrates the direct influence of the oxidizing agent on the oxide layer of the stainless steel.

## 4. Conclusions

(i) The contact area between the indenter and the vertical position of the material increases with increasing oxidizer content. The size of the scratch (width and depth) is proportional to the concentration of the oxidizing agent, and the material removal of stainless steel during the scratching process is in the form of plastic domain removal.
(ii) The analysis of nano-scratch depth–displacement–load curves revealed that the maximum depth of the oxide layer was achieved at an oxidant concentration of 8%. At this concentration, the obtained scratch depth and initial transition pressure were measured at 2153 nm and 58.47 mN, respectively.
(iii) The reaction mechanism of stainless steel was elucidated from both electrochemical corrosion and reaction kinetics perspectives, demonstrating that ferric chloride, acting as an oxidizing agent, effectively catalyzes redox reactions on stainless steel surfaces. Additionally, the elastic–plastic transformation during the scribing process and XRD test results indicate changes in the physical phase of the stainless steel surface layer and an increase in peak intensity. These findings serve as evidence that electrochemical corrosion induces grain refinement in the surface layer of stainless steel.

The impact of oxidizer concentration on the softening layer of 304 stainless steel holds significant importance. The thickness of the oxide layer plays a crucial role in determining the efficiency and surface quality of ultra-precision machining. However, factors influencing the thickness of the oxide layer are diverse and complex, warranting further in-depth research in subsequent studies.

## Figures and Tables

**Table 1:** The main chemical composition of 304 stainless steel.

Main Elements	Cr	Mn	Fe	Ni
Mass%	19.70	1.88	70.66	7.76

**Table 2:** Stainless steel pre-treatment grinding process parameters.

Contents	Revolution	Pressure	Time	Particle Size
Parameter	60 r/min	13.17 kPa	20 min	1 μm (Al_2_O_3_)

## Data Availability

Data are contained within the article.

## References

[1] Bellamkonda P., Dwivedy M., Sudersanan M., Visvalingam B. (2024). Microstructural characteristics and properties of wire arc additive manufactured 304L austenitic stainless steel cylindrical components by different arc welding processes. Mater. Chem. Phys..

[2] Liu Y., Bai L., Pham S., Wang J., Wan S. (2024). Temperature dependence of energy dissipation during nanoscale wear of AISI 304L stainless steel. Wear.

[3] Chen J., Peng Y., Wang Z., Lv F. (2023). Influence of Fenton-like reactions between hydrogen peroxide and ferric chloride on chemical mechanical polishing 304 stainless steel. Int. J. Adv. Manuf. Technol..

[4] Liu T., Li J., Wei C., Li Z., Jiang W., Qin W., Duan Y., Mao Q., Wang Z., Mao J. (2024). Formation mechanisms of heterostructures in 304L stainless steel processed by cold rolling and annealing. Vacuum.

[5] Chen J., Sun T., Su J., Li J., Zhou P., Peng Y., Zhu Y. (2021). A novel agglomerated diamond abrasive with excellent micro-cutting and self-sharpening capabilities in fixed abrasive lapping processes. Wear.

[6] Peng Y., Wang Z., Fu Y., Zhang X., Chen J. (2022). An interactive water lubrication mechanism of γ-LiAlSi_2_O_6_ glass-ceramics in friction and wear. Wear.

[7] Wang Y., Jiang L., Qian L. (2019). Effect of hydrogen peroxide on chemical mechanical polishing performance of bearing steel. Light Ind. Mach..

[8] Zhang D., Liu J., Chen Y., Wang M., Ge X. (2015). Investigation on S-136 steel surface planarization by chemical mechanical polishing. Microelectron. Eng..

[9] Weng J., Lin R., Rong X. (2020). Study on polishing slurry of hydrogen peroxide-oxalic acid in CMP 304 stainless steel. MATEC Web Conf..

[10] Wu H., Jiang L., Zhong X., Liu J., Qin N., Qian L. (2020). Exploring the role of −NH2 functional groups of ethylenediamine in chemical mechanical polishing of GCr15 bearing steel. Friction.

[11] Andreatta F., Lanzutti A., Vaglio E., Totis G., Sortino M., Fedrizzi L. (2019). Corrosion behaviour of 316L stainless steel manufactured by selective laser melting. Mater. Corros..

[12] Detriche S., Vivegnis S., Vanhumbeeck J.F., Felten A., Louette P., Renner F.U., Delhalle J., Mekhalif Z. (2020). XPS fast depth profile of the native oxide layers on AISI 304, 316 and 430 commercial stainless steels and their evolution with time. J. Electron Spectrosc. Relat. Phenom..

[13] Lodhi M.J.K., Deen K.M., Greenlee-Wacker M.C., Haider W. (2019). Additively manufactured 316L stainless steel with improved corrosion resistance and biological response for biomedical applications. Addit. Manuf..

[14] Revilla R.I., Wouters B., Andreatta F., Lanzutti A., Fedrizzi L., De Graeve I. (2020). EIS comparative study and critical Equivalent Electrical Circuit (EEC) analysis of the native oxide layer of additive manufactured and wrought 316L stainless steel. Corros. Sci..

[15] Wang B., Zhang Z., Chang K., Cui J., Rosenkranz A., Yu J., Lin C.T., Chen G., Zang K., Luo J. (2018). New Deformation-Induced Nanostructure in Silicon. Nano Lett..

[16] Zhang Z., Guo D., Wang B., Kang R., Zhang B. (2015). A novel approach of high speed scratching on silicon wafers at nanoscale depths of cut. Sci. Rep..

[17] Cheng Q., Wang Y. (2022). Effect of Surface Scratches on Corrosion Behavior of 304 Stainless Steel Beneath Droplets of Solution (0.5 mol/L NaCl + 0.25 mol/L MgCl_2_). J. Chin. Soc. Corros. Prot..

[18] Kuromoto N.K., Fiusa D.L., Cantão M.P., Lepienski C.M. (1999). Nanoscratching characterization of austenitic stainless steel modified by cathodic hydrogenation. Mater. Sci. Eng..

[19] Goel S. (2014). The current understanding on the diamond machining of silicon carbide. J. Phys. D Appl. Phys..

[20] Bifano T.G., Dow T.A., Scattergood R.O. (1991). Ductile-regime grinding: A new technology for machining brittle materials. J. Eng. Ind..

[21] Pathote D., Jaiswal D., Singh V., Behera C.K. (2022). Optimization of electrochemical corrosion behavior of 316L stainless steel as an effective biomaterial for orthopedic applications. Mater. Today Proc..

[22] Zeng H., Yang Y., Zeng M., Li M. (2021). Effect of dissolved oxygen on electrochemical corrosion behavior of 2205 duplex stainless steel in hot concentrated seawater. J. Mater. Sci. Technol..

[23] Lee S., Han J.-H., Lee S.-H., Baek G.-H., Park J.-S. (2018). Review of organic/inorganic thin film encapsulation by atomic layer deposition for a flexible OLED display. Miner. Met. Mater. Soc..

[24] Sugimoto A., Ochi H., Fujimura S., Yoshida A., Miyadera T., Tsuchida M. (2004). Flexible OLED displays using plastic substrates. IEEE J. Sel. Top. Quantum Electron..

[25] Danilov F.I., Bogdanov D.A., Smyrnova O.V., Korniy S.A., Protsenko V.S. (2022). Electrodeposition of Ni–Fe alloy from a choline chloride-containing ionic liquid. J. Solid State Electrochem..

[26] Su J., Wang Y., Wang Z., Li Y., Ma L., Pang M. (2021). Study on chemical action mechanism of ferric chloride-based polishing slurry in CMP of 304 stainless steel. J. Inst. Eng. Ser. E.

[27] Ferreira E.A., Noce R.D., Fugivara C.S., Benedetti A.V. (2011). Evaluation of 316L stainless steel corrosion resistance in solution simulating the acid hydrolysis of biomass. J. Electrochem. Soc..

[28] Zamanizadeh H.R., Sunde S., Pollet B.G., Seland F. (2022). Tailoring the oxide surface composition of stainless steel for improved OER performance in alkaline water electrolysis. Electrochim. Acta.

